# Some Ocular Paralyses

**Published:** 1904-11-12

**Authors:** 


### The SOME OCULAR PARALYSES.

An ocular paralysis is a clinical event which can hardly escape attention. Even though it be of so minor a degree that the resulting defect in the movement of the eyeball is too slight to engage the recognition of the casual observer, the patient himself is forced to give heed by the serious annoyance and inconvenience produced by the consequent diplopia. A very slight want of harmony between the movement of the two eyeballs is sufficient to cause images of external objects to be formed on non-corresponding parts of the retinas, and therefore to produce the development of double vision. Hence it is a sound clinical rule which urges attention to a complaint of double vision even though no obvious disturbance in the ocular movements can be appreciated by the physician. No doubt in any case of suspected malingering the patient must be subjected to tests which may lead him into inconsistent statements, but if this discipline is adopted with negative results double vision must be allowed, even though no definite ocular paresis can be appreciated. The use of a coloured glass placed before one eye, or perhaps still better of Maddox's rod, together with a lamp or candle that can be carried into various parts of the visual field, will lead fairly easily to a recognition of the muscle which is at fault. From this, to a reference to the corresponding nerve supply is a brief and elementary step, which even the tyro in anatomy is prepared to take. But further advance in the diagnosis is often attended with much uncertainty. Whether the lesion is in the nerve trunk or in the nerve centres is a question not always easy of solution, and of not less difficulty in many instances is the attempt to present a pathological as well as an anatomical diagnosis. The muscle, the nerve or nerve centre at fault, are more or less on the surface, but the nature of the lesion is frequently a matter of mere speculation. In the case of the sixth nerve even the anatomical suggestion is far from having a precise value. The long course of the nerve and its immediate relationship to the base of the skull render it liable to interruption by indirect causes; even a tumour so remote as the cerebral convolutions may lead to a paralysis of one or both sixth nerves.

### Rheumatic Paralysis.

Perhaps the first point bearing on the ætiological question is whether an ocular paralysis can be produced by exposure to cold or by some agency more or less correctly spoken of as "rheumatic." In so many instances the paresis is found to depend directly or indirectly on syphilis that the student fresh from the confident atmosphere of the out-patient department is apt to look scornfully on a less precise interpretation. Yet the weight of authority and experience is decidedly in favour of the view that the nerves supplying the ocular muscles may be damaged by a process essentially similar to that which in the case of the facial nerve causes Bell's paralysis and which is associated with the influence of cold or damp. There is no pathological evidence in favour of this opinion, but the authority of many experienced clinical observers upholds it. For the most part, paralyses so explained are confined to one nerve, or to one or possibly neighbouring branches of a single nerve. Gowers,[1] however, has described three cases in which all the nerves supplying the muscles of one eye were affected, and at the same time also the optic nerve. In reference to these "rheumatic" cases it may be noted that they often persist over weeks or months, and therefore this element in a case of ocular paralysis is not of necessity and by itself proof of the existence of a gross intracranial lesion.

### Tabes Dorsalis and Disseminated Sclerosis.

Next to cold or rheumatism the suggestion to which an unaccompanied ocular paralysis gives rise is probably tabes dorsalis. Without question such a paralysis may be the first clinical event in the evolution of this disease. And it is of much importance in relation to the statement just made regarding the possible duration of a "rheumatic" paralysis to note that in tabes dorsalis the paralysis is often of brief duration and tends to recur. Other things being equal, an obstinate persistence of the paralysis is much more suggestive of rheumatic than of tabetic causation. The former, too, is invariably unilateral in distribution, and in the majority of cases this is true also of tabes, but occasionally in this disease paralyses are seen on both sides, as, for example, in cases of double ptosis. Another chronic nervous disease which may be heralded or accompanied by an ocular paralysis is disseminated sclerosis, and here also the paresis may have but a temporary duration. Among the rarer causes of ocular paralyses are diphtheria and diabetes, the lesion in each case being presumably a peripheral neuritis. Considering the frequency with which diphtheria produces paralysis of the ciliary muscle, and occasionally also of the sphincter iridis, it is somewhat remarkable that it so seldom attacks the nerve supply of the external muscles of the eyeball.

### Ocular Paralyses and Intra-Cranial Disease.

At various times cases have been recorded which extend the clinical associations of ocular paralyses in more or less unusual directions and some of these may here be conveniently summarised. Professor de Lapersonne[2] describes a case of otitis media, of acute origin and confirmed by the escape of pus on incision of the tympanic membrane, which was followed by vertical and homonymous diplopia. There were no other symptoms suggestive of intracranial disease and the patient made a good recovery. A similar case has been recorded by Pechin.[2] In neither of these instances could there have been any serious cerebral or cerebellar disease and the suggestion has been advanced that the paralysis was a reflex result of irritation of the vestibular nerve. The significant practical point, therefore, is that an ocular paralysis may apparently arise in disease of the middle ear without the occurrence of any intracranial complication, or at least of any complication demanding surgical interference. This is all the more important seeing that exactly the same statement has been made with regard to optic neuritis. This view has received countenance from no less an authority than Sir Wm. Gowers,[3] and in the discussion of a clinical record by Mayo Collier,[4] it would appear to find considerable justification. At first sight no two events could be more directly suggestive of serious disease within the skull than an ocular paralysis and double optic neuritis, and occurring in a case of middle-ear disease they might well be regarded as urging the surgeon to operative interference. That, indeed, was the position in which Mayo Collier found himself in reference to his patient. Yet, after full consideration, he refrained from opening the skull and the result appeared to justify his decision. It seems therefore necessary to admit that, in some cases at all events, middle-ear disease may produce both an ocular paralysis and double optic neuritis, and that these two events in such a case do not necessarily mean intracranial abscess or other serious complication within the skull. Another example of an ocular paralysis apart from intracranial disease is found in a case reported by Chas. P. Emerson[5] in which the paresis occurred comparatively early in the course of enteric fever. It is suggested by way of explanation that the condition was due to a limited meningitis. An alternative view is that the paresis of the nerve depended on thrombosis in one of the cerebral veins or sinuses and this would appear to receive some support from the fact that at a later date thrombosis occurred in one of the femoral veins.

### Hysteria.

A very unusual case of ocular paralysis is that reported by Edgar Stevenson.[6] The patient was a girl of 15 years and there was almost complete paralysis of all the ocular muscles except the internal recti. Considerable improvement took place after four applications of the high-frequency currents, and the case, on the whole, was regarded as of a functional nature. It would be of interest to know the later developments in this patient. The suggestion of hysteria as an explanation of an ocular paralysis must always be under suspicion until the lapse of time has shown it to be well-founded, and this criticism must apply to the case just mentioned. A better-established instance is that described by Leslie Buchanan.[7] The patient was a woman of 35 years. Whilst in her usual health she developed a paralysis of the right external rectus muscle. This persisted for some little time, and then gradually disappeared. Shortly afterwards, however, a corresponding paralysis appeared on the left side, and this also had but a temporary duration. The patient was free from all signs of organic nervous disease, and there was no history or evidence either of rheumatism or syphilis. Further, for two years after the occurrence of the paralysis she continued in good health, and there had been no relapse. Of course, even the lapse of two years does not exclude absolutely the possibility of developments in the nervous system. Still, on the whole, the case does suggest caution before dogmatically asserting that an ocular paralysis cannot possibly be other than a note of organic disease. Myasthenia must also be mentioned among the rarer causes of defect in the ocular movements.[8] This disease may also cause ptosis and weakness of the orbicularis palpebrarum. The symptoms are bi-lateral, and there are the other evidences of the disease, such as the ready exhaustion of muscular energy in walking, chewing, swallowing, etc.

### Anomalous Cases.

Whilst it is common enough to have an ocular paralysis affecting only, in one direction or other, the lateral movements of the eyeball, restriction of the paralysis to movement in the vertical plane are decidedly uncommon. A case reported by Simeon Snell[9] is an exception to this rule. The patient was a man of 50 years who appeared to have become quietly unconscious after taking his evening meal. On waking in the morning he complained of pain in the head and of double vision. Examination showed inability to move either eye upwards though the movements in all other directions were perfect. After some weeks the upward excursion of the eyeballs had been completely regained. Such a record seems to suggest a limited vascular intracranial lesion, though the exact situation of this is not easily surmised. Possibly a small hæmorrhage involving the corresponding nerve centres on each side of the middle line is the most probable explanation. Another example of a restriction of an ocular paralysis to a limited area is seen in a case reported by Leonard Guthrie.[10] The patient was a woman aged 28, and was, in all probability, the subject of syphilis. There were various other evidences of organic nervous disease, but, so far as the musculature of the eyeballs was concerned, the only abnormal conditions were dilatation and inaction to light of the left pupil, with paresis of the left superior rectus muscle. Mr. W. Beaumont[11] has described fleeting ocular paralysis in cases of rheumatoid arthritis, and in one such case recorded by J. W. Malim[12] all the ocular movements are said to have been limited. In the case just mentioned there were muscular tremors and some degree of Von Graefe's sign, but without exophthalmos, goitre, or tachycardia. This is of interest in relation to the occasional announcement of an ocular paralysis occurring in the course of Graves' disease.[13] Such authorities as Bristowe and Jendrassik, among others, have recorded examples of this association. The paralysis may involve only a single muscle, or there may be a complete ophthalmoplegia, and this latter has been present on both sides. B. W. Gowring[14] has recently described the occurrence of a complete ophthalmoplegia in a case of pertussis. The paralysis not only affected the recti and oblique muscles, but also the levator palpebrarum, the ciliary muscle, and the sphincter iridis on each side. It may be supposed that the child in straining during a paroxysm of coughing ruptured a small blood-vessel in the immediate neighbourhood of the nerve nuclei. Another suggestion is that the paralysis was comparable to that in infantile paralysis, and depended on a toxin affecting the nerve centres. The first of these two suggestions receives some support from the fact that the onset of the paralysis was very sudden and also from the well-known experience that subconjunctival and even subcutaneous hæmorrhages are by no means uncommon in pertussis. There are also reasons for believing that monoplegias, hemiplegias, and other paralyses which occasionally complicate this disease depend on cerebral or spinal softenings the result of hæmorrhages produced presumably by the increased blood pressure which attends the paroxysmal cough.

### Migraine.

A very important practical association of ocular paralysis is with migraine. Some of the cases recorded appear to have been pure cases of migraine, and of a purely functional nature; the attacks have occurred at intervals over a long series of years, each being accompanied by double vision and ocular paralysis and the patient between the attacks being in good health. It is, however, certain, that in some cases which seem to be of this order, there is definite organic disease. Thus Gowers[15] describes the case of a woman who suffered from childhood from periodical attacks of palsy of the left third nerve and who died of phthisis pulmonalis at 30 years of age. At the post-mortem examination the roots of the nerve were found to be surrounded by small grey granulations which contained tubercle bacilli. Dr. James Taylor[16] has also had experience of hemicrania with ocular paralysis. He doubts whether these cases are true migraine and, whilst allowing that the headaches in the two groups is similar, doubts whether the ocular paralysis ever occurs with such migrainous symptoms as hemianopsia, fortification lines, etc. It is certain that in some instances periodical attacks of headache developing in childhood have sooner or later been complicated by a recurring ocular paralysis. For a time the paralysis has, like the headache, disappeared for considerable periods. But sooner or later it has proved permanent, and in some instances the presence of a tumour has been demonstrated in the course of the nerve trunk. Presumably, at first, such a lesion does not interrupt the passage of nerve currents unless reinforced by a local congestion associated with the attack of hemicrania. Gradually under the influence of such attacks, and perhaps due to the growth of the tumour, the obstruction becomes complete and permanent, and a persisting paralysis is thus produced.

A survey of the cases here summarised seems to show that the clinical incidents they relate are decidedly exceptional. They are all of interest, but they hardly touch the field of ocular paralyses as these are seen in daily experience. For such incidents one must, of course, be prepared, but the practical clinical rule is hardly affected by them. That rule may be stated to be that an uncomplicated ocular paralysis throws suspicion on the nervous system, and that only the lapse of time and the negative result of continued and systematic observation can cause such a suspicion to be dispelled.

1 Dis. of Nerv. System, vol. ii. 2 L'Echo Med. du Nord. June 2, 1901 (quoted in the Med. Rev., Sept. 1901). 3 Trans. of Ophthalmological Soc., 1902, p. 282. 4 Trans. of Brit. Laryngological and Rhinological Assoc., 1901, p. 46. 5 Johns Hopkins Hosp. Reports, 1900, vol. viii, p. 897. 6 Brit. Med. Jour., Jan. 25, 1902. 7 Trans. of Glas. Medico-Chirurgical Soc., vol. i., p. 275. 8 Brit. Med. Jour., May 24 and 31, 1902. 9 Ibid., Nov. 22, 1902. 10 Lancet, Oct. 25, 1902. 11 Lancet, Feb. 6, 1904 12 Brit. Med. Jour., Feb. 14, 1903. 13 Lancet, Aug. 13, 1904. 14 Brit. Med. Jour, Dec. 26, 1903. 15 Dis. of Nerv. System, vol. ii., p. 181. 16 Brit. Jour. of Children's Dis., Jan. 1904.

